# Diagnostic performance of direct wet mount microscopy in detecting intestinal helminths among pregnant women attending ante-natal care (ANC) in East Wollega, Oromia, Ethiopia

**DOI:** 10.1186/s13104-018-3380-z

**Published:** 2018-05-04

**Authors:** Hylemariam Mihiretie Mengist, Gebreselassie Demeke, Olifan Zewdie, Adugna Belew

**Affiliations:** 1 Department of Medical Laboratory Sciences, College of Health Sciences, Debre Markos University, P.O. Box: 269, Debre Markos, Ethiopia; 2 Department of Medical Laboratory Sciences, College of Health Sciences, Wollega University, P.O. Box: 395, Nekemte, Ethiopia

**Keywords:** Diagnostic performance, Direct microscopy, Helminths, Pregnant women

## Abstract

**Objective:**

The aim of this study was to evaluate the diagnostic performance of direct wet mount microscopy compared to formalin ether concentration (FEC) technique in detecting intestinal helminths in pregnant women.

**Results:**

The total prevalence of intestinal helminths was 18.8% (70/372) by direct wet mount microscopy and 24.7% (92/372) by FEC technique (P < 0.001). The sensitivity, negative predictive value (NPV) and test efficiency (TE) of direct wet mount microscopy in diagnosing intestinal helminths was 76, 92.7 and 94%, respectively. The sensitivity of direct w

et mount microscopy was very low in detecting ova of *Hymenolepis nana*. The two methods showed excellent agreement in detecting ova of Hook worm and *Ascaris lumbricoides* (Kappa > 0.81) but they fairly agreed in detecting ova of *Hymenolepis nana* (Kappa = 0.39). Intestinal helminths were underdiagnosed and the total diagnostic performance of direct wet mount microscopy was significantly poor in detecting intestinal helminths as compared to FEC technique. Routine use of FEC method is recommended for the diagnosis of intestinal helminths in pregnant women.

## Introduction

Intestinal parasitic infections, especially due to helminths, increase anemia in pregnant women. The results of this are low pregnancy weight gain and intra uterine growth retardation, followed by low birth weight, with its associated greater risks of infection and higher prenatal mortality rates. An estimated 44 million pregnant women have hookworm infections which can cause chronic loss of blood from the intestines and predisposes the women to developing iron deficiency anemia [1].

Although several diagnostic tools are available to diagnose intestinal helminths, direct wet mount microscopy is commonly used for the diagnosis of intestinal parasitic infections generally in Africa and particularly in Ethiopia [2–5]. However, low sensitivity of the direct wet mount technique has been reported to have poor performance in the detection of low intensity infection elsewhere which shows that the use of direct wet mount microscopy will significantly increase misdiagnosis of intestinal helminthic infections [6].

Different studies showed that formol-ether concentration technique (FEC) is more sensitive than the conventional direct wet mount microscopy. Therefore, the employment of FEC techniques as a confirmatory test in routine laboratory examination of stool will significantly aid in accurate determination and management of parasitic infections [7].

In Ethiopia, especially in health center and hospital laboratories, diagnosis of intestinal helminths solely depends on direct wet mount microscopy which is not reliable [7]. For better follow up and make morbidity free pregnancy, screening of pregnant women for intestinal helminths should be at most sensitive. The present study is, therefore, aimed to evaluate the diagnostic performance of direct wet mount microscopy among pregnant women using FEC technique as a gold standard.

## Main text

### Materials and methods

#### Study setting and context

A cross sectional study was conducted in selected five health centers of East Wollega Zone of Oromia region, Ethiopia namely Jimma- Arjo health center, Arjo-Gudatu health center, Sire health center, Gute health center and Nekemte health center between November 2015 and January 2016.

#### Sample size and sampling technique

Sample size was calculated using single population proportion formula considering 95% CI and a marginal error of 0.05 as follows;

$${\text{n}} = {\text{Z}}_{{\left( {{\text{a}}/2} \right)^{2} }} {\text{P}}\left( {1 - {\text{P}}} \right)/{\text{d}}^{2}$$

where n is sample size which is 372; P is prevalence of intestinal helminths in pregnant women from previous similar study which was 0.41 [8]; d is marginal error which is 0.05.

Finally, 372 pregnant women were consecutively enrolled from the five health centers using proportional stratified sampling.

#### Study population and data collection

Pregnant women taking anti-helminthic/anti-protozoan drugs or received mass drug administration within the past 2 weeks were excluded. Medical laboratory professionals were trained on data collection for this particular study to attain standardization and reliability. All reagents used were checked for their expiry date and prepared according to the manufacturer’s instructions. We obtained all the reagents from Pharmaceuticals Fund and Supply Agency of Ethiopia.

#### Laboratory methods

A single stool specimen was collected from each participant. Freshly voided stool specimens were directly examined microscopically and preserved with 10% formalin for further analysis. Then preserved specimens were processed using formalin-ether concentration technique and examined microscopically for ova and larvae of intestinal helminths.

##### Direct wet mount analysis

A singles stool specimen was obtained from all study participants. Then a direct saline wet mount microscopy of each sample was used to detect intestinal parasites microscopically. Briefly, one drop of normal saline was added on a clean slide and then a stool equivalent to a match stick head (2 mg) was mixed with it. Then, the wet mounts were examined under light microscope under 100X and 400X magnifications [9].

##### Formol–Ether concentration (FEC) method

A portion of each preserved stool specimen was taken and processed following standard procedures. Briefly, 1 gm stool was placed in a clean conical centrifuge tube containing 7 mL 10% formol water by using applicator stick. The resulting suspension was filtered through a sieve into another conical tube. After adding 3-4 ml of diethyl ether to the formalin solution, the content was centrifuged at 3200 rpm for 1 min. The supernatant was discarded; smear was prepared from these sediment and observed under light microscope with a magnification of 100X and 400X after air dried [9].

#### Data quality assurance

All laboratory analyses were carried out using standard operating procedures. Only one medical laboratory technologist performed the direct wet mount microscopy in each health center and one senior medical laboratory technologist performed the FEC technique blindly for all specimens.

#### Operational definitions

- False positive (FP)–the individual does not have the condition but tests positive for the condition.
- Sensitivity—is the probability that a truly infected individual will test positive (sensitivity = TP/(TP + FN)).
- Specificity—is the ability of a method to identify non-infected individuals correctly (specificity = TN/(TN + FP)).
- Positive predictive value (PPV): the probability that those testing positive by the method are truly infected (PPV = TP/(TP/FP)).
- Negative predictive value (NPV)–the probability that those testing negative by the test are truly uninfected (NPV = TN/(TN + FN)).
- Test efficiency (TE)–the overall ability of the test to correctly identify positives from negatives and implies the absence of false positives& false negatives (TE = (TP + TN)/(TN + TP + FN + FP)).
- Accuracy: closeness of the tests to a true value (a true positive or a true negative).

#### Statistical analysis

Data entry and analysis were done using SPSS version 20 statistical software for descriptive and inferential statistics. Prevalence, sensitivity, specificity, positive and negative predictive values of direct wet mount microscopy was determined by using FEC as a gold standard technique. Agreement of the two methods in detecting intestinal helminths was determined by Kappa test. Kappa values were interpreted as follows; from 0.01 to 0.20 as slight agreement, from 0.21 to 0.40 as fair agreement, 0.41–0.60 as moderate agreement, 0.61–0.80 as substantial agreement and 0.81–0.99 as perfect agreement [10].

### Results

#### Prevalence of intestinal helminths

The total prevalence of intestinal helminths using direct wet mount microscopy and formol-ether concentration (FEC) method was 18.8% (70/372) and 24.7% (92/372), respectively with the difference rate of 5.9% (P < 0.001) (Table 1).

**Table 1:** Distribution of intestinal helminths and agreement of direct wet mount microscopy and FEC method among pregnant women attending ANC in East Wollega, Oromia, Ethiopia from November 2015 to January 2016

Intestinal helminths	Prevalence detected by each method				Kappa value	P value
	Direct wet mount microscopy (N = 372)		FEC method (N = 372)			
	Number	Percent	Number	Percent		
Total intestinal helminths	70	18.8	92	24.7	0.81	< 0.001
Mixed infection	2	0.54	4	1.08	ND	< 0.001
Hook worm	48	12.9	56	15.1	0.84	< 0.001
*Ascaris lumbricoides*	20	5.37	24	6.5	0.88	< 0.001
*Hymenolepis nana*	2	0.54	6	1.61	0.39	< 0.001
*Taenia species*	NPF	–	5	1.34	ND	NA
*Strogyloides stercoralis*	NPF	–	1	0.27	ND	NA

*NPF* no parasite found, *NA* not available, *ND* not determined

Regarding the detection of specific intestinal helminths, direct wet mount microscopy reported 12.9% (48/372) Hookworm, 5.37% (20/372) *Ascaris lumbricoides,* and 0.54% (2/372) *Hymenolepis nana*. But *Taenia* species and *Strogyloides stercoralis* were not detected by this method. Mixed infection was reported in two (0.54%) of study participants using this method unlike the FEC technique which detected mixed infections in four (1.08%) of pregnant women. Based on FEC technique, the prevalence rate of Hookworm, *Ascaris lumbricoides*, *Hymenolepis nana*, *Taenia* species and *Strogyloides stercoralis* was 15.1% (56/372), 6.5% (24/372), 1.6% (6/372), 1.34% (5/372) and 0.27% (1/372), respectively. The two methods showed significantly different diagnosing ability in detecting intestinal helminths (P < 0.001) (Table 1). Comparatively the detecting ability of direct wet mount microscopy was poor in diagnosing Hookworm, *Hymenolepis nana* and *Taenia* species specifically.

#### Sensitivity and negative predictive value (NPV) of direct wet mount microscopy

The sensitivity of direct wet mount method in detecting total intestinal helminths, Hookworm, *Ascaris lumbricoides* and *Hymenolepis nana* was 76, 85.7, 83.3 and 33.3%, respectively. *Taenia* species and *Strongyloides stercoralis* were not detected by the direct wet mount microscopy (Table 2). This result indicates that direct wet mount microscopy is not enough to rule out absence of intestinal helminths in a single stool specimen in general.

**Table 2:** Sensitivity, NPV and TE of direct wet mount microscopy in detecting intestinal helminths among pregnant women attending ANC in East Wollega, Oromia, Ethiopia from November 2015 to January 2016

Direct wet mount microscopy	FEC (N = 372)		Sensitivity 95% CI	NPV 95% CI	TE 95% CI
	True Positive	True Negative			
Total intestinal helminths					
Positive	70	NFP	76% 0.69–0.82	92.7% 0.89–0.95	94% 0.91–0.96
Negative	22	280			
Hook worm					
Positive	48	NFP	85.7% 0.83–0.88	97.5% 0.94–0.99	97.8% 0.95–0.99
Negative	8	316			
*Ascaris lumbricoides*					
Positive	20	NFP	83.3% 0.80–0.86	98.8% 0.96–0.99	98.9% 0.97–0.99
Negative	4	348			
*Hymenolepis nana*					
Positive	2	NFP	33.3% 0.29–0.38	98.9% 0.97–0.99	98.9% 0.97–0.99
Negative	4	366			
*Taenia species*					
Positive	NPF	NFP	NA	98.6% 0.94–0.99	98.6% 0.94–1
Negative	5	367			
*Strogyloides stercoralis*					
Positive	NPF	NFP	NA	99.7% 0.97–1	99.7% 0.97–1
Negative	1	371			

*NPF* no parasite found, *NA* not available, *NFP* no false positive

The specificity and positive predictive value of direct wet mount microscopy was 100% due to absence of falsely positive result. The ability of direct wet mount to rule out true negatives as negative was better (above 92.7%) in general. The probability of truly negative pregnant women to be negative by direct wet mount microscopy for the presence of Hookworm, *Ascaris lumbricoides, Hymenolepis nana, Taenia species and Strongyloides stercoralis* was 97.5, 98.8, 98.9, 98.6 and 99.7%, respectively (Table 2).

#### Test efficiency (TE) of direct wet mount microscopy

The overall ability of direct wet mount microscopy to correctly diagnose intestinal helminths (TE) was 94%. The test efficiency of this method in diagnosing specifically Hookworm was comparatively low (97.8%). Direct wet mount microscopy showed similar efficiency in diagnosing *Ascaris lumbricoides* and *Hymenolepis nana* (98.9%) (Table 2).

#### Agreement of the test methods

The two methods perfectly agreed in diagnosing total intestinal helminths, ova of Hookworm and *Ascaris lumbricoides* (Kappa > 0.81). But these methods fairly agreed in detecting ova of *Hymenolepis nana* (Kappa = 0.39) (Table 1).

### Discussion

World Health Organization (WHO) recommends the use of Kato-Katz method in duplicate slides and other techniques like FEC and McMaster methods for the detection of human soil transmitted helminths (STH) like *Ascaris lumbricoides*, *Trichuris trichiura* and the Hookworms. All of these techniques rely on visual examination of a small sample of stool to determine the presence and number of STH eggs with different sensitivities especially in low transmission areas [11–14].

The traditional direct wet mount microscopy has lower sensitivity in detecting intestinal helminths when compared to the FEC method. Therefore, the use of FEC technique as a confirmatory test in routine laboratory examination of stool will significantly aid in accurate determination and management of parasitic infections [7].

The findings of the present study showed that direct wet mount microscopy has showed lower prevalence rate of intestinal helminths when compared to the FEC method (18.8% versus 24.7%) (P < 0.001) which is similar to a study done in Nigeria [15]. These differences in diagnostic performance might be due the low sensitivity of the direct wet mount technique as indicated by other studies, inter personal skill variations or could be due to the technical errors of the two methods. This means direct wet mount is not enough to rule out the absence of intestinal helminths as reported by other studies [16]. This low detection ability of direct wet mount microscopy in single stool specimen could be due to intermittent shading of intestinal helminths in a single stool and low ova production ability of some intestinal helminths.

Direct wet mount microscopy showed significantly low detecting ability specifically in diagnosing Hookworm*, Ascaris lumbricoides, Hymenolepis nana* and *Taenia species* in the present study. This is similar with reports from other studies which stated as direct wet mount has lower sensitivity in detecting *Ascaris lumbricoides*, *Schistosoma mansoni*, *Trichuris trichiura* and Hookworms [7]. The lower sensitivity in this study could be due to inclusion of only a single stool specimen which impedes the identification of intermittently shading intestinal helminths.

The sensitivity of direct wet mount microscopy was generally low in diagnosing total intestinal helminths (76%) and very low in detecting ova of *Hymenolepis nana* (33.3%) in the present study. The sensitivity of direct wet mount in detecting intestinal helminths in this study is higher than a study done in Ethiopia [17] which showed a sensitivity of 48.9%. This could be due to the inclusion of all intestinal parasites in the previous study and inter personal skill variations.

Although the two methods had a perfect agreement in detecting total intestinal helminths similar to other studies [17], they fairly agreed in detecting *Hymenolepis nana* which means that direct wet mount microscopy is poor in detecting low ova producing helminths from a single stool specimen.

Our findings showed that direct wet mount microscopy exhibited low sensitivity for the detection of intestinal helminths as compared to the FEC technique which is similar with another study done in Ethiopia [18]. This suggested that the use of direct wet mount microscopy alone in diagnosing intestinal helminthic infections is insufficient and may lead to false negative results.

### Conclusion

The prevalence of intestinal helminths was underreported by direct wet mount microscopy. The total diagnostic performance of direct wet mount microscopy for the diagnosis of intestinal helminths in pregnant women was significantly low compared to FEC technique in this study. Therefore, the FEC method should be used as a routine technique in health center or hospital laboratories for the diagnosis of intestinal helminths especially in pregnant women who need special emphasis.

## Limitations

This study has limitations regarding using sensitive techniques as a true gold standard to compare laboratory techniques and collection of only a single stool specimen which may impede the diagnostic performance of the methods.

## Abbreviations

ANC: antenatal care
FEC: formalin ether concentration
TP: true positive
TN: true negative
FP: false positive
FN: false negative
NPV: negative predictive value
PPV: positive predictive value
TE: test efficiency
